# Semiconducting properties of layered cadmium sulphide-based hybrid nanocomposites

**DOI:** 10.1186/1556-276X-6-523

**Published:** 2011-09-06

**Authors:** Zoraya López-Cabaña, Clivia Marfa Sotomayor Torres, Guillermo González

**Affiliations:** 1 Faculty of Sciences, Universidad de Chile, P.O. Box 653, Santiago, Chile; 2 Centre of Development of Nanoscience and Nanotechnology (CEDENNA) Santiago, Chile; 3 Catalan Institute of Nanotechnology (ICN-CIN2), Campus UAB Edifici CM7, 08193-Bellatera, Barcelona, Spain; 4 Catalan Institute for Research and Advanced Studies (ICREA), 08010 Barcelona, Spain; 5 Department of Physics, Universidad Autónoma de Barcelona, 081093 Bellaterra, Barcelona, Spain

## Abstract

A series of hybrid cadmium salt/cationic surfactant layered nanocomposites containing different concentrations of cadmium sulphide was prepared by exchanging chloride by sulphide ions in the layered precursor CdX*_x_*(OH)*_y_*(CnTA)*_z _*in a solid phase/gas reaction, resulting in a series of layered species exhibiting stoichiometries corresponding to CdS*_v_*X*_x_*(OH)*_y_*(CnTA)*_z_*, constituted by two-dimensional CdCl_2_/CdS ultra-thin sheets sandwiched between two self-assembled surfactant layers. The electronic structure of CdS in the nanocomposite is similar to that of bulk, but showing the expected features of two-dimensional confinement of the semiconductor. The nanocomposite band gap is found to depend in a non-linear manner on both the length of the hydrocarbon chain of the surfactant and the concentration of the sulphide in the inorganic sheet. The products show photocatalytic activity at least similar and usually better than that of "bulk" CdS in a factor of two.

## Introduction

During the last years, much effort has been invested on the development of strategies to assemble inorganic nanoparticles in well-defined arrays. In order to obtain technologically useful nanocrystal-based materials, their spatial orientation and arrangement need to be taken into account, in addition to the size and shape of the nanocrystal and their surface chemistry [1].

Numerous synthetic methods leading to semiconductor nanocrystalline II-VI materials, for example, CdS, ZnS, PbS and/or CdSe, have been reported where a number of templates have been used for forming and/or stabilising the nanoparticles, among them are mesoporous materials [2,3], dendrimers [4], polymers [5,6] or surfactants [7,8]. Mesophases of lyotropic liquid crystals have been also used to produce CdS nanocrystals [9,10]. In general, it is common to find organic-inorganic nanostructured composites which provide a rich source of new materials with promising technological applications [11]. However, reports on layered arrangements of semiconductors like CdS are still scarce in literature. Among these, a method to obtain lamellae and dendrites of ZnS, starting from the layered precursor ZnS·(NH_2_CH_2_CH_2_NH_2_)_0.5_, has been reported [12], and cadmium chalcogenides in the solid state, i.e. with S, Se or Te, containing two ethylendiamine molecules per Cd atom, have also been prepared [13]. In these products, the presence of amine avoids the structural collapse and helps to form the corresponding metal chalcogenide.

In this work we describe the synthesis of a series of layered single phases in which different amounts of CdS are confined in a CdCl_2 _matrix. The optical and photocatalytic properties of these nanocomposites are studied, as well as the dependence of the latter with the concentration of sulphur in the samples. It is found that these nanocomposites have better photocatalytic activity than "bulk" CdS.

## Experimental section

### Materials and chemicals

Cadmium chloride hydrated (Aldrich, Sigma-Aldrich Chemie GmbH, Steinheim, Germany), hexadecyltrimethylammonium bromide (CTAB, SigmaUltra 99%, Sigma-Aldrich, St. Louis, USA), 99%), octadecyltrimethylammonium bromide (OTAB, Aldrich, 97%, Sigma-Aldrich Chemie GmbH, Steinheim) (CTAB and OTAB abbreviated as CnTA with *n *= 16 or 18, respectively), iron(II) sulphide (Merck, Merck KGaA, Darmstadt, Germany) and hydrochloric acid (Merck) were used as received. Gaseous hydrogen sulphide was prepared in place by reaction of iron(II) sulphide with HCl 37% *v*/*v *and washed with deionised water.

### Preparation of CdX*_x_*(OH)*_y_*(CnTA)*_z _*nanocomposites

The CdCl_2_-CnTA complex was synthesised by treating a solution of the salt with the aqueous gel of the surfactant CTAB or OTAB. Typically, to the surfactant gel about 4 mol% in water, a CdCl_2 _ethanol solution was added in 0.05 M concentration to obtain a molar ratio CdCl_2_/surfactant of 1:2. The suspension was vigorously stirred at a temperature of 60°C for 24 h. The solid product was extracted by centrifugation, washed several times with ethanol, dried under vacuum and stored under argon. Elemental chemical analyses and stoichiometries are given in Table 1.

**Table 1:** Elemental analyses and stoichiometric formulae of precursor, Cd_1_X*_x_*(OH)*_y_*(CnTA)*_z_*, and nanocomposites, Cd_1_S*_v_*X*_x_*(OH)*_y_*(CnTA)*_z_*

Solid	Elemental analysis (%)					Stoichiometric formula^c^
	N^a^	C^a^	H^a^	S^a^	Cd^b^	
CdCl_2_-CTAB	3.05 (3.13)	50.20 (50.99)	10.99 (10.13)	0	11.16 (10.92)	Cd_1_X_3.5_(OH)_0.8_(C_19_H_42_N)_2.3_**·**3H_2_O
	2.57 (2.50)	40.67 (40.66)	8.48 (8.12)	6.62 (6.57)	8.98 (10.53)	Cd_1_S_0.54_X_2.58_(OH)_0.24_(C_19_H_42_N)_1.9_·6.3H_2_O
	2.21 (2.16)	35.00 (35.15)	7.07 (6.76)	8.03 (8.05)	7.75 (7.87)	Cd_1_S_0.93_X_2.25_(OH)_0.09_(C_19_H_42_N)_2.2_·2.9H_2_O
CdCl_2_-CTAB + H_2_S	2.10 (2.05)	33.33 (33.34)	6.80 (6.49)	9.21 (9.22)	9.44 (9.96)	Cd_1_S_0.95_X_1.7_(OH)_0.05_(C_19_H_42_N)_1.65_·3.6H_2_O
	2.03 (1.95)	31.81 (31.81)	6.36 (6.05)	9.36 (9.34)	8.62 (10.70)	Cd_1_S_0.95_X_0.74_(OH)_0.09_(C_19_H_42_N)_0.73_·0.8H_2_O
	1.18 (1.16)	18.21 (18.92)	3.17 (3.51)	12.51 (13.00)	18.21 (19.03)	Cd_1_S_0.99_X_0.18_(C_19_H_42_N)_0.16_

CdCl_2_-OTAB	2.83 (2.85)	51.53 (51.46)	11.24 (10.15)	0	11.73 (11.45)	Cd_1_*X*_3.2_(OH)_0.8_(C_21_H_46_N)_2_·3H_2_O

	2.42 (2.37)	42.65 (42.68)	9.10 (8.65)	6.96 (6.95)	3.99 (3.25)	Cd_1_S_0.58_X_1.49_(OH)_0.29_(C_21_H_46_N)_0.94_·4.6H_2_O
	2.26 (2.21)	39.71 (39.78)	8.49 (7.88)	7.54 (7.54)	5.34 (5.33)	Cd_1_S_0.62_X_0.99_(OH)_0.32_(C_21_H_46_N)_0.55_·1.9H_2_O
CdCl_2_-OTAB + H_2_S	1.94 (1.89)	33.97 (33.95)	7.10 (6.73)	8.97 (8.93)	6.74 (6.45)	Cd_1_S_0.71_X_0.83_(OH)_0.22_(C_21_H_46_N)_0.47_·2.1H_2_O
	1.86 (1.84)	32.83 (33.14)	6.77 (6.38)	9.79 (9.90)	7.34 (6.58)	Cd_1_S_0.73_X_0.79_(OH)_0.21_(C_21_H_46_N)_0.46_·0.9H_2_O
	1.75 (1.72)	30.92 (30.97)	6.28 (6.01)	10.00 (10.00)	7.38 (6.55)	Cd_1_S_0.97_X_0.51_(OH)_0.08_(C_21_H_46_N)_0.53_·1.6H_2_O

^a ^± 0.30%; ^b ^± 0.10%; ^c^*X *= *y*Cl^- ^+ (1 - *y*)Br^-^.

### Preparation of CdS*_v_*X*_x_*(OH)*_y_*(CnTA)_z _nanocomposites

The metallic sulphur-surfactant nanocomposites were prepared by bubbling gaseous H_2_S through an ethanol suspension of the precursor CdX*_x_*(OH)*_y_*(CnTA)_z_. The reaction was kept at room temperature under constant stirring for a period of 2, 4, 8, 16 or 24 h. The yellow solids obtained were separated by centrifugation, washed twice with ethanol, dried under vacuum and stored under argon. Analyses are reported in Table 1. CdS used as control sample along this work, named "bulk" CdS, was prepared from CdCl_2 _under the same conditions used for preparing the nanocomposites.

### Characterization

Fourier transformed infrared (FT-IR) spectra (4,000-500 cm^-1^) were recorded on a Bruker IFS 25 model infrared spectrophotometer (Bruker Optik GmbH, Ettlingen, Germany). Samples for FT-IR were prepared using pressed KBr disc technique. Raman vibrational spectra were performed on a Bruker Raman Fourier transform spectrometer RFS 100/S (Bruker Optik GmbH, Ettlingen, Germany). The samples grinded in agate mortar were put into sealed capillary glass tubes to be placed in the sample holder of the instrument. The beam of a Nd:YAG laser (*λ *= 1064 nm) was used as excitation source. X-ray powder diffraction analysis was performed using a Siemens D5000 diffractometer (Siemens company, Karlsruhe, Germany) with Cu Kα radiation (1.5418 Å, operation voltage 40 kV, current 30 mA). The morphology of the products was examined by scanning electron microscopy (SEM) using an S-5000 field-emission SEM (Hitachi Ltd., Japan),, operating at beam voltages between 1 and 10 kV. The chemical composition of the samples was determined by elemental chemical analysis (PerkinElmer 240C PerkinElmer Inc., California, USA) and atomic absorption spectrometry (Unicam 929, Agilent Technologies, USA).

### Optical measurements

Diffuse reflectance UV-visible (UV-vis) spectra were recorded using a Shimadzu UV-vis spectrophotometer, double beam model 2450 PC, equipped with an integrating sphere (Shimadzu Co., Tokyo, Japan. Barium sulphate was used in all cases as reference material. Spectra were recorded in the range of 200 to 800 nm at room temperature. Reflectance measurements were converted to absorption spectra using the Kubelka-Munk function [14].

The photoluminescence (PL) spectra were recorded at room temperature using a PerkinElmer spectrofluorometer, LS 55 model (PerkinElmer Inc., California, USA). This spectrometer is equipped with a 150-W Xenon lamp source, emission and excitation monochromator configurations and a photomultiplier tube (R-106). The spectral response was virtually flat in the examined spectral regions. Analysis of PL spectra was performed by deconvoluting the spectra by fitting experimental data points to a sum of *n *Gaussian functions using Origin 6.0 multi-peak fitting package; confidence criterion was adjusted *R*-square and reduced Chi-square values.

### Photocatalytic properties

The photocatalytic activity of CdS-surfactant nanocomposites was tested using, as reaction model, the photodegradation of methylene blue. Experiments were performed typically using 50 ml of a 2 × 10^-5 ^M aqueous solution of the dye and photocatalyst loadings in the range of 0.5 to 0.6 g/L. This solution was irradiated with a UV-visible light source emitting in the 270 to 310 nm range. The change in the concentration of methylene blue in the solution while irradiating was monitored by measuring the absorbance at regular intervals between 30 and 240 min using the UV-visible spectrometer mentioned in "**Optical measurements"**.

## Results and discussion

### Structure of cadmium sulphide-surfactant nanocomposites

The nature of the CdCl_2 _crystal structure, in which bonding along the crystallographic *c*-axis is relatively weak, easily leads to mesostructured layered products in the presence of cationic surfactants [15]. This feature was exploited in this work using the layered CdCl_2_/cationic surfactant as a precursor of the corresponding mixed CdCl_2_/CdS derivatives by specifically direct exchange of chloride by sulphide atoms in a solid phase/gas reaction. Given the high affinity of cadmium-ion for soft Lewis bases like sulphide, exchange reaction occurs spontaneously, and exothermically, under rather mild conditions. Thus, the layered structure of the precursor, as discussed below, remains practically unaltered. Varying the amount of added hydrogen sulphide, the relative concentration of sulphur in the sample may be regulated in the range of 6.62 to 12.51 atom% (see Table 1) without disrupting the pristine structure of the solid. In the following, we analyse the properties of products with the highest concentration of sulphur, i.e. in the range of 0.54 to 0.99 atoms of sulphur per cadmium ion.

**Figure 1:**
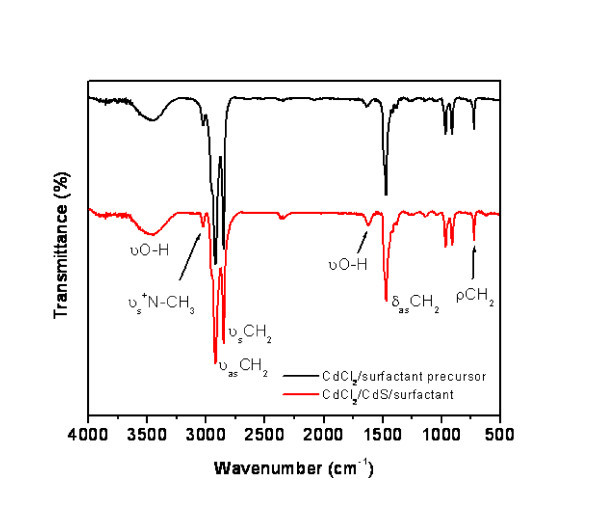
**FT-IR spectra of precursor, Cd_1_X_3.5_(OH)_0.8_(C_19_H_42_N)_2.3_·3H_2_O) (red), and lamellar nanocomposite (Cd_1_S_0.99_X_0.18_(C_19_H_42_N)_0.16_)) (black)**. CdCl_2_/surfactant precursor (Cd_1_X_3.5_(OH)_0.8_(C_19_H_42_N)_2.3_·3H_2_O) (red line) and the CdCl_2_/CdS/surfactant lamellar nanocomposite (Cd_1_S_0.99_X_0.18_(C_19_H_42_N)_0.16_) (black line).

**Figure 2:**
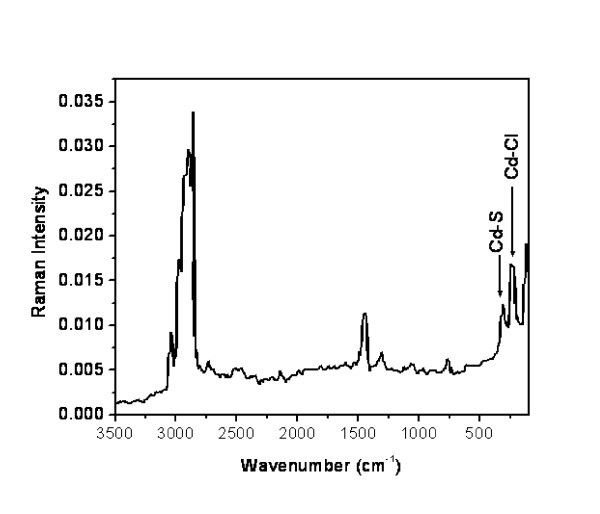
**FT-Raman spectrum of the CdCl_2_/CdS/surfactant lamellar nanocomposite (Cd_1_S_0.99_X_0.18_(C_19_H_42_N)_0.16_)**.

FT-IR spectra show that the composition and structure of the surfactant in both CdCl_2_/surfactant and CdCl_2_/CdS/surfactant lamellar nanocomposites are similar. Typical spectra of CdS nanocomposites and of its precursor are compared in Figure 1. Characteristic bands of the -CH_2_ group in the surfactant - symmetric and asymmetric stretching centred around 2,851 and 2,920 cm^-1^, respectively; asymmetric scissor deformation around 1,470 cm^-1 ^and the rocking at about 7,222 cm^-1 ^[16] - as well as the symmetric stretching vibrations of surfactant head group ^+^N-CH_3 _around 3,024 cm^-1 ^remain practically unaltered after sulphur intake. The mixed nature, Cd-Cl and Cd-S bonds, of the inorganic sheets may be appreciated in the Raman spectrum of the CdCl_2_/CdS/surfactant nanocomposite being displayed in Figure 2, where the stretching vibrations assigned to Cd-Cl (236 cm^-1^) bonds [17] and the 1LO phonon frequency for Cd-S (307 cm^-1^) are clearly detected [18].

**Figure 3:**
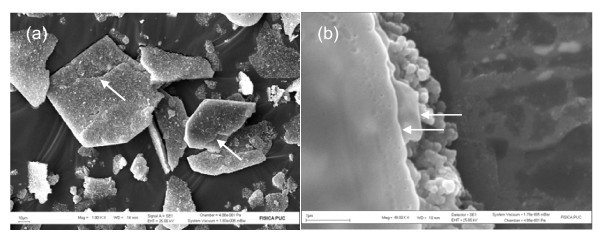
**SEM images of as-synthesised Cd_1_S_0.95_X_1.7_(OH)_0.05_(C_19_H_42_N)_1.65_·3.6H_2_O nanocomposite**. (**a**) General view with size bar indicating 10 μm and (**b**) zoomed view with size bar indicating 1 μm.

Electron microscopy shows that the morphology of resulting CdS nanocomposites corresponds to a lamellar structure as shown in Figure 3. It is seen that the nanocomposite (Cd_1_S_0.95_X_1.7_(OH)_0.05_(C_19_H_42_N)_1.65_·3.6H_2_O) forms plates of about 94 × 141 μm^2 ^with a rather homogeneous surface with steps characteristic of graphite-like structures (Figure 3a). At higher resolution, the images show *quasi*-spherical grains in the border of the plates which appear to coalesce forming the lamellae (Figure 3b). This behaviour is widely known in small-size lamellar structures, which spontaneously tend to form closed spherical or tubular structures to minimise surface energy. The morphology of these nanocomposites is totally different than that of "bulk" CdS.

**Figure 4:**
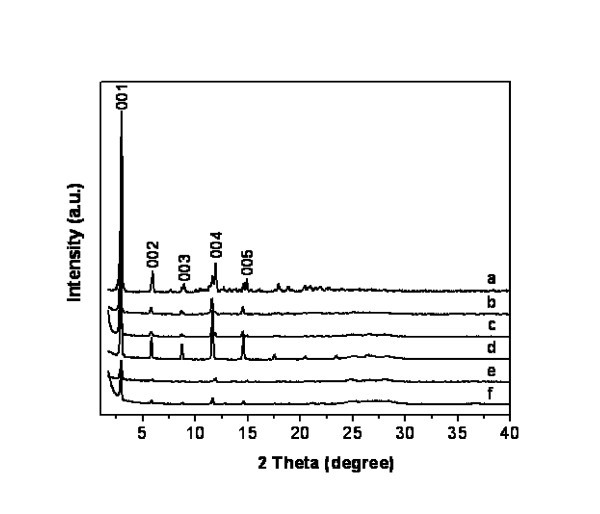
**X-ray diffraction patterns of (a) CdCl_2_/surfactant precursor and CdCl_2_/CdS/surfactant nanocomposites at different sulphurisation times**. (b) 2 h, (c) 4 h, (d) 8 h, (e) 16 h and (f) 24 h.

**Figure 5:**
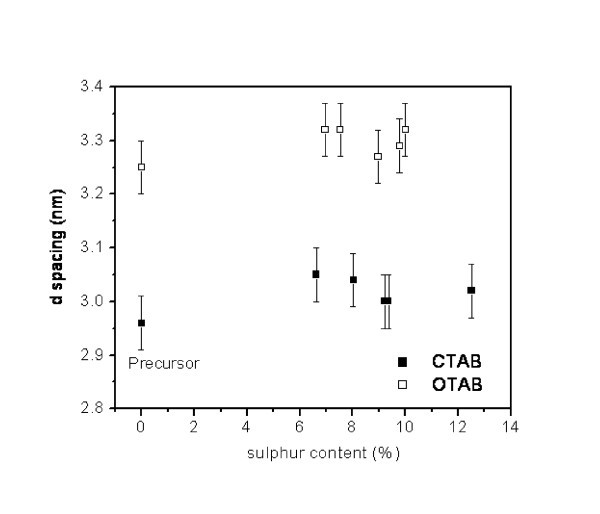
**X-ray diffraction inter-lamellar d-spacing as function of sulphur content for CdCl_2_/CdS/surfactant nanocomposites**.

X-ray diffraction analysis of the products independently corroborates the lamellar morphology of these nanocomposites. Diffraction patterns illustrated in Figure 4 correspond to selected series of nanocomposites with different sulphur contents. All diffraction patterns exhibit a series of reflections at low angles characteristic of well-ordered layered species. Indexation of observed Bragg reflections yields inter-planar distances which follow the relationship (*d*_1_/*d*_1_) = 1, (*d*_1_/*d*_2_) = 2, (*d*_1_/*d*_3_) = 3, etc, thus pointing to layered structures ordered in the direction perpendicular to the [00l] planes. Moreover, the diffraction patterns indicate that all nanocomposites, independently of their sulphur content, are pure phases; thus, no phase segregation is apparently taking place in these systems. As shown graphically in Figure 5, inter-lamellar distances observed for nanocomposites clearly correlate with the length of the hydrocarbon chain in the surfactant. However, for a given surfactant, they do not vary significantly with sulphur content, being in the range of 3.00 to 3.07 nm and 3.27 to 3.32 nm for derivatives with CTAB and OTAB, respectively. These values are close, but not identical, to those observed when chlorides are used as precursors. Indeed, the incorporation of sulphur produces a slight increment of inter-lamellar distances, which varies between 0.02 and 0.09 nm along the series.

### Diffuse reflectance measurements

Absorption spectra, *F*(*R*_∞_), of the CdS-surfactant nanocomposites were obtained from diffuse reflectance spectroscopy using the Kubelka-Munk function (Equation 1):

(1) $$F\left(R_{\infty }\right)=\frac{K}{S}=\frac{{\left(R_{\infty }-1\right)}^{2}\;}{2R_{\infty }}$$

where *R*_∞ _is the diffuse reflectance, and *K *and *S *are the absorption and diffusion coefficients, respectively, and *R*_∞ _< 1 if *K *≠ 0.

**Figure 6:**
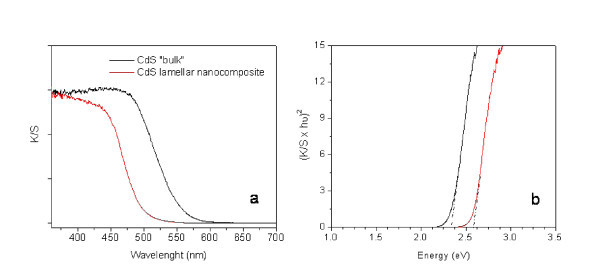
**Reflectance diffuse Kubelka-Mulk spectra (a) and linear fitting considering direct band gap transitions (b)**. (**a**) Reflectance diffuse spectra (after Kubelka-Mulk transformation) of "bulk" CdS and CdCl_2_/CdS/surfactant lamellar nanocomposite (Cd_1_S_0.99_X_0.18_(C_19_H_42_N)_0.16_) and (b) plot (*K*/*S *× hυ)^2 ^against hυ showing the linear fitting to the main linear segment of the curve considering a direct band gap transition.

Derived absorption spectra of the nanocomposites are similar to that of "bulk" CdS but show blue-shifted absorption band edges (Figure 6a). This is interpreted as confinement of electronic states of the semiconductor, as expected for this kind of structures. The corresponding band gaps were evaluated using the standard relationship given in the Equation 2:

(2) $$\alpha (\upsilon )=A{(h\nu -\text{Eg)}}^{m\text{/2}}$$

where *α *is the absorption coefficient, Eg is the band gap energy and *m *= 1 for allowed direct transitions. Since *α*(*υ*) is proportional to *K*/*S*, the band gap can be obtained from the plot (*F*(*R*_∞_) × *hv*)^2 ^against *hv*.

**Figure 7:**
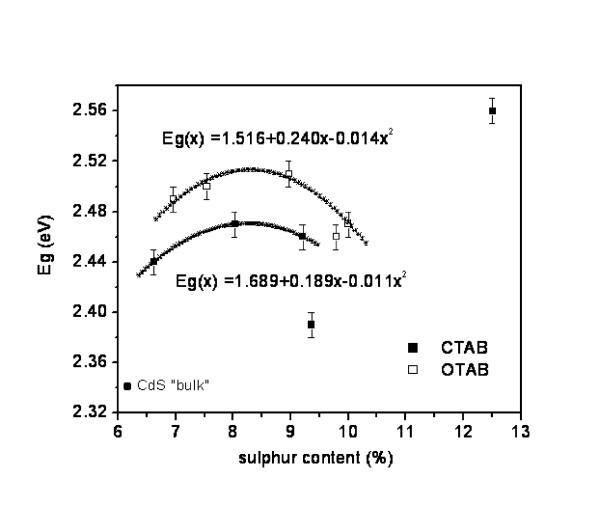
**Optical band gap energy of the nanocomposites Cd_1_S*_v_*X*_x_*(OH)*_y_*(C*_n_*H_2*n*+1_N)*_z_***. As a function of sulphur content and surfactant (CTAB or OTAB).

The band gap energies of the nanocomposites, calculated as explained above from plots like that illustrated in Figure 6b, are listed in Table 2. They are consistently higher than the energy band gap of "bulk" CdS, with increments in the range of 0.05 to 0.22 eV. The dependence of the band gap on sulphur content is plotted in Figure 7. A clear non-linear relationship is observed fitted by an empirical polynomial relationship indicated in the same figure. This feature may be interpreted, in first approximation, as arising from two types of confinement. One of them is probably due to the separation between the semiconductor layers, which is mainly determined by the length of the hydrocarbon chain in the surfactant. The second one could be the result of interactions of the semiconductor across the layer. However, this interpretation, particularly the second one, needs to be further investigated. Experiments as well as calculations to shed more light on this problem are in progress. Nevertheless, from a practical point of view, based on the systems studied here, the possibility to design CdS nanocomposites with a predetermined band gaps has been demonstrated.

**Table 2:** Band gap energies of "bulk" CdS and lamellar CdS/surfactant nanocomposites synthesised for this work

Compound	Sulphur content (%)	Eg ± 0.01 (eV)
CdS "bulk" (control sample)	-	2.34 (theoretical value, 2.42) [19]
Cd_1_S_0.54_X_2.58_(OH)_0.24_(C_19_H_42_N)_1.9_·6.3H_2_O	6.62	2.44
Cd_1_S_0.93_X_2.25_(OH)_0.09_(C_19_H_42_N)_2.2_·2.9H_2_O	8.03	2.47
Cd_1_S_0.95_X_1.7_(OH)_0.05_(C_19_H_42_N)_1.65_·3.6H_2_O	9.21	2.46
Cd_1_S_0.95_X_0.74_(OH)_0.09_(C_19_H_42_N)_0.73_·0.8H_2_O	9.36	2.39
Cd_1_S_0.99_X_0.18_(C_19_H_42_N)_0.16_	12.51	2.56
Cd_1_S_0.58_X_1.49_(OH)_0.29_(C_21_H_46_N)_0.94_·4.6H_2_O	6.96	2.49
Cd_1_S_0.62_X_0.99_(OH)_0.32_(C_21_H_46_N)_0.55_·1.9H_2_O	7.54	2.50
Cd_1_S_0.71_X_0.83_(OH)_0.22_(C_21_H_46_N)_0.47_·2.1H_2_O	8.97	2.51
Cd_1_S_0.73_X_0.79_(OH)_0.21_(C_21_H_46_N)_0.46_·0.9H_2_O	9.79	2.46
Cd_1_S_0.97_X_0.51_(OH)_0.08_(C_21_H_46_N)_0.53_·1.6H_2_O	10.00	2.47

### Photoluminescence emission-excitation spectroscopy

**Figure 8:**
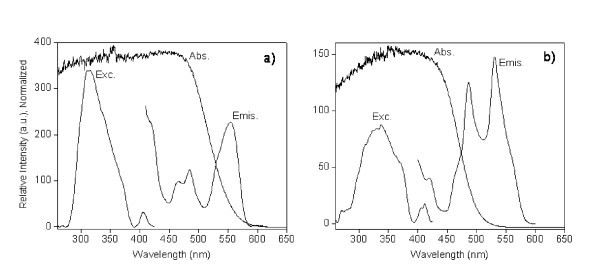
**Absorption, excitation and emission spectra of "bulk" CdS (a) and of the nanocomposite (b)**. Absorption (Abs), photoluminescence emission (Emis, *λ*_PL _= 350 nm) and excitation (Exc, *λ*_detec _= 490nm) spectra of (**a**) "bulk" CdS and (**b**) Cd_1_S_0.71_X_0.83_OH_0.22_(C_21_H_46_N)_0.47_·2.1H_2_O nanocomposite.

Absorption, excitation and emission spectra of "bulk" CdS and of the nanocomposite Cd_1_S_0.71_X_0.83_OH_0.22_(C_21_H_46_N)_0.47_·2.1H_2_O (Eg = 2.51 eV) are compared in Figure 8.

**Figure 9:**
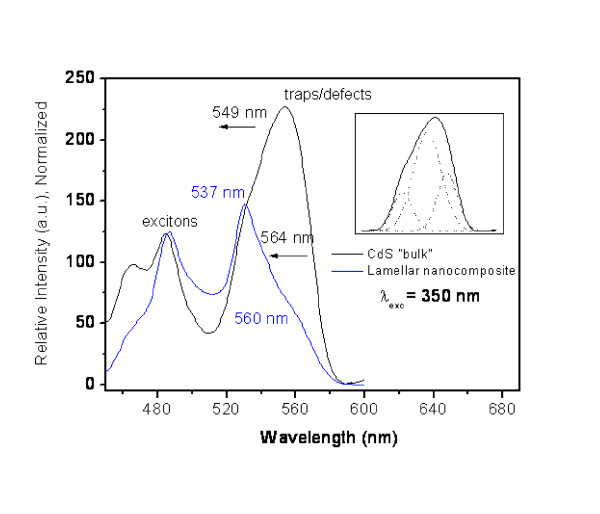
**Photoluminescence emission spectra of "bulk" CdS and lamellar nanocomposite Cd_1_S_0.71_X_0.83_OH_0.22_(C_21_H_46_N)_0.47_·2.1H_2_O**. Insert: Main band in emission spectra deconvoluted into three Gaussian curves. *R*^2 ^= 0.99974, Chi^2 ^= 1.58983.

Emission spectra of both species, Figure 9, show three bands in the range of 400 to 500 nm, centred at 422 nm (2.94 eV), 464 nm (2.67 eV) and 485 nm (2.56 eV). These are attributed to emissions near the absorption edge. A band at 550 nm is also observed which may correspond to emission involving surface states or traps associated to interfacial crystalline defects [19]. In the case of CdS, this emission is associated to sulphur and cadmium atoms vacancies [20]. Comparing the exciton bands in both species, no significant relative shift is observed. However, in the band near 550 nm, a slight blue shift in the spectra of the nanocomposites is detected. This may be even better observed by deconvoluting the main band in emission spectra into three Gaussian curves (*R*^2 ^= 0.99974, Chi^2 ^= 1.58983) as shown in the insert in Figure 9.

**Figure 10:**
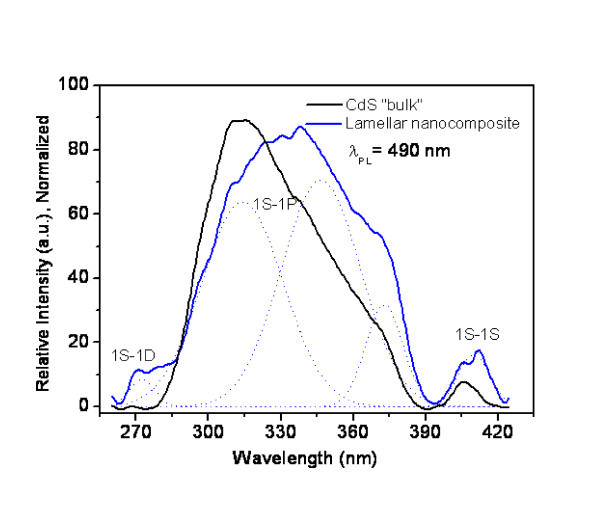
**Photoluminescence excitation spectra of "bulk" CdS and lamellar nanocomposite Cd_1_S_0.71_X_0.83_OH_0.22_(C_21_H_46_N)_0.47_·2.1H_2_O**. As measured and deconvoluted into to five Gaussian curves. *R*^2 ^= 0.99819, Chi^2 ^= 1.77748.

Excitation spectra of both "bulk" CdS and nanocomposite are dominated by a broad band between 260 and 430 nm (Figure 10). Analysis of excitation and PL spectra, performed by deconvoluting the spectra by fitting experimental data points to a sum of Gaussian functions (*R*^2 ^= 0.99819, Chi^2 ^= 1.77748), reveals the existence of three and five bands, respectively. This five PL bands probably correspond to interband transitions. Indeed for "bulk" CdS, following assignations have been proposed: the band at 299 nm (4.15 eV) to the transition 1S-1D; the bands at 314 nm (3.95 eV), 341 nm (3.64 eV) and 369 nm (3.36 eV) to transitions 1S-1P; and the band at 407 nm (3.05 eV) to exciton transition 1S-1S [21]. In the nanocomposites, these values are similar to those in "bulk" CdS. So these bands appear to be characteristic of transitions in semiconductor itself, being not altered by the shape of the particles or the existence of interfaces.

### Photocatalytic properties

The photocatalytic activity of CdS nanocomposites was tested using methylene blue as a model compound, which has been proved to be appropriate to study such processes [22]. Complete decolouration accompanies photodegradation, thus permitting easy spectrometric determination of the degradation process and its irreversible character. The use of this dye is favourable since the regeneration of colour by oxygen or other oxidant is avoided. Our experiments were performed in aqueous media, and the progress of dye photodegradation was followed by observing the characteristic dye absorption peak intensity centred at 664 nm.

**Figure 11:**
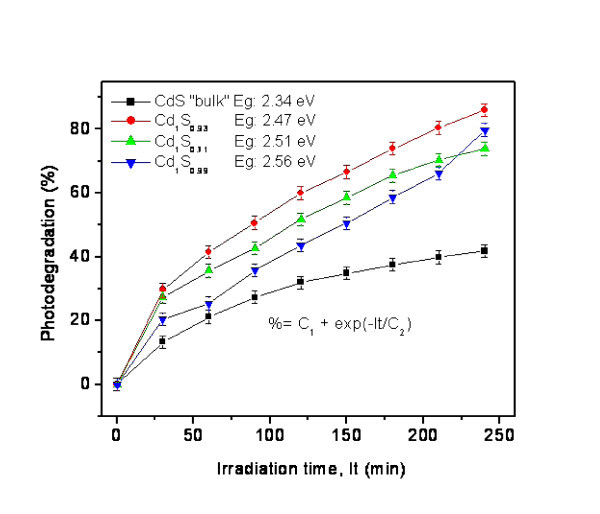
**Photodegradation of methylene blue using CdS "bulk" and CdCl_2_/CdS/surfactant lamellar nanocomposites**. With different sulphur contents as catalysts.

Figure 11 shows the results of photodegradation of methylene blue and "bulk" CdS obtained by using a series of nanocomposites with different sulphur content as catalyst. The photodegradation occurs exponentially as a function of irradiation time, pointing to comparable degradation mechanisms in all cases. Interestingly, the nanocomposites appear to be more efficient as photocatalysts than "bulk" CdS, although the actual amount of semiconductor in the nanocomposites is lesser than in "bulk" CdS because of the higher molecular weight of the former due to the presence of about 45% of organic material. An explanation of the enhanced catalytic activity displayed by the nanocomposites is probably due to two factors. The larger surface area available, given its nanostructured nature, and the hydrophobicity of the particle surface would permit a better interaction with the dye. The preliminary studies described here are not sufficient to clarify this issue at present. Additional work considering different types of surfactants is planned.

## Conclusions

Results described in this paper show that by using adequate synthesis procedures, it is possible to obtain hybrid semiconducting cadmium sulphide nanocomposites in which CdS forms part of two-dimensional ultra-thin inorganic sheets sandwiched between two self-assembled surfactant layers. These nanostructures, containing predetermined amount of CdS, are found in bulk held together by van der Waals interactions, thus generating layered graphitic-like structures with inter-laminar distances which correlate well with the hydrocarbon chain length of the surfactant. The electronic structure of Cd, as deduced from absorption, excitation and emission spectra, is similar to that of bulk but shows "all" the features expected for a two-dimensional confinement of the semiconductor. The CdS band gap may be, to some extent, regulated by selecting both the length of the hydrocarbon chain of the surfactant and the concentration of the sulphide in the layers. The charge transfer ability of the nanocomposites, evaluated from the photocatalytic activity of these products, appears to be better than that of "bulk" cadmium sulphide. These results are encouraging in the search of methods to design and prepare tailor-made novel functional semiconducting materials.

## Abbreviations

CTAB: hexadecyltrimethylammonium bromide; Eg: band gap energy; OTAB: octadecyltrimethylammonium bromide; SEM: scanning electron microscopy.

## Competing interests

The authors declare that they have no competing interests.

## Authors' contributions

ZLC conceived the study, did the experimental analyses and participated in drafting the manuscript. CMST and GG conceived the study and participated in its design, drafting and coordination.
